# Place, the Built Environment, and Means Restriction in Suicide Prevention

**DOI:** 10.3390/ijerph16224389

**Published:** 2019-11-10

**Authors:** Nathaniel J. Pollock

**Affiliations:** 1 School of Public Health, University of Alberta, Edmonton, AB T6G 2R3, Canada; nathaniel.pollock@med.mun.ca; 2 Division of Community Health and Humanities, Faculty of Medicine, Memorial University, St. John’s, NL A1C 5S7, Canada

**Keywords:** suicide, means restriction, built environment, place, surveillance, epidemiology

## Abstract

Restricting access to lethal means is a key public health intervention for preventing suicide. Means restriction research has often focused on suicide methods that are modifiable through legislation or policy interventions. However, some of the most common methods such as hanging may not be sensitive to regulation. The aims of this paper are to examine built environment and place-based approaches to means restriction in suicide prevention, and further consider the connections between place, the environment, and suicide methods. To increase knowledge about specific methods and mechanisms of injury in suicide deaths, higher resolution data for surveillance and epidemiology is required. Data that can be used to better discern patterns about specific locations and materials used in suicide and self-harm will support efforts to uncover new directions for prevention.

## 1. Background

Restricting access to lethal means is a key public health intervention for preventing suicide [1]. Research in this area has often focused on legislation and policy interventions that modify access to suicide methods such as firearms, pesticides, and domestic gas [2,3]. Limiting the availability of these methods is effective in reducing both the method-specific incidence and overall suicide mortality rates because restricting access to one method does not necessarily lead to substitution for an alternative [2,4]. Conversely, the rate of suicide by hanging is not sensitive to regulation or other universal approaches as the materials used are varied and ubiquitous in most households [5]. In light of this, it was encouraging to read the recent paper by Kariippanon and colleagues on their proposal for advancing knowledge to prevent suicide from hanging by ceiling fans [6]. 

The authors rightly pointed out that evidence gaps about suicide by hanging are due in part to the lack of high resolution data on specific means and anchor points [6]. They proposed that modifying the design of anchor points such as ceiling fans could decrease the lethality of suicide attempts by hanging [6]. These are novel considerations for work in this area, and ones which may be complemented by other lines of inquiry. The aims of this paper are to briefly examine built environment and place-based approaches to means restriction interventions in suicide prevention, and propose additional work in epidemiology and surveillance that further consider the connections between place, the environment, and suicide methods.

## 2. The Built Environment and Means Restriction

Means restriction interventions may not always explicitly consider the built environment and place, yet these are underlying dimensions in public health approaches to suicide prevention. In part, this is because the methods that are most commonly implicated in suicide deaths are often related to what is easily available in a given setting or context [1]. This helps explain some of the variation in the prevalence of suicide methods across sex, time, setting, and country [1,4,7,8]. In institutions such as prisons and hospitals, removing ligatures and anchor points can reduce the number of hanging deaths [2,9]. Similarly, creating structural barriers and encouraging help-seeking at ‘hotspots’ such as bridges or train stations is an effective way to modify public spaces to increase safety and prevent suicide by jumping [9,10,11]. These are two ways that restricting access to lethal means have addressed the relationships between those at risk of suicide and physical spaces in communities and cities. 

A persistent challenge for built environment approaches to means restriction is that more than 75% of suicide deaths occur at home [12] rather than in institutions or public spaces which can be more directly regulated or designed [13]. Despite this, policy interventions can affect household access to lethal means such as poisons. In the United Kingdom for example, reducing the toxicity of domestic gas during the mid-20th century led to a reduction in suicide rates [3]. Likewise, legislation to reduce the package size of over-the-counter pain medication in 1998 resulted in a 22% reduction in suicide deaths due to analgesic poisoning [13]. 

Relatedly, there is increasing recognition of the importance of place in understanding the uneven distribution of risk factors and in harnessing local assets for the development of context-specific interventions. Measuring geospatial variations in suicide and self-harm is one of the ways that suicide prevention has begun to appreciate differences between neighborhoods, communities, and regions [14,15,16]. The differences that such analyses uncover can provide valuable insight for service delivery and policy, and can inform “place-based” interventions in suicide prevention that are focused on redressing rate disparities and health inequity [14,17,18].

## 3. Place-Based Approaches to Suicide Prevention

Place-based approaches are an important part of the evidence in suicide prevention [9]. Typically, they involve community-wide and multi-level interventions in a defined geographic area. In several contexts, such interventions have significantly reduced suicide attempts, especially those with highly lethal means [19], and suicide deaths [20]. In the Circumpolar North, the global region that includes the northern areas of eight Arctic countries, place-based interventions are a fundamental aspect of suicide prevention in Indigenous communities [21]. A major part of the value of these types of approaches are that they are rooted in community strengths, informed by local knowledge and culture, and often address overlapping priorities related to mental health and social equity [22]. From this context, there are several examples of means restriction interventions that integrate dimensions of place and the built environment.

Firearms are a leading cause of suicide death in Alaska and rates of firearm-related suicide are elevated for Alaska Native peoples compared to non-Indigenous populations [23]. In a randomized controlled trial to asses the impact of safe firearm storage, households in six Alaska Native communities were chosen at random to receive a lockable gun cabinet in their home [23]. This group was compared to households that were waitlisted for the gun cabinets. The purpose was to assess gun and ammunition storage practices before the intervention and at 12 and 18 months afterwards. At baseline, 93% of households had unlocked guns and 89% had unlocked ammunition [23]. Twelve months after gun cabinets were installed, 35% households in the intervention group had unlocked guns compared to 89% of households in the wait list control group, and rates of unlocked ammunition were 34% and 85% respectively [23]. The study showed that an environmental modification improved safe firearm storage in households, and therefore modified a proximal risk factor for suicide. However, it was not clear if the changes were sustained over the long term, nor was the impact on suicide attempts or deaths specifically assessed. 

In Nunavut, an Inuit territory in northern Canada, guns are commonplace in many households because they are used for subsistence hunting. Yet, in contrast to Alaska, firearm-related injuries account for 16% of suicide deaths; suicide by hanging is the predominant method [24]. Following a suicide cluster in one community, local leaders recognized that many of the suicide deaths occurred in bedroom closets in social housing units, where closet rods were the main ligature points [25]. The community started a means restriction initiative that involved removing the closet rods and bedroom locks [25]. Although the impact of changes to the f was not reported, conceptually, this case study and the study from Alaska both resonate with themes in this discussion.

## 4. Implications for Suicide Epidemiology and Surveillance

In the examples from Alaska and Nunavut, the interventions were an effort to disrupt socially patterned methods of self harm. These approaches were possible because the communities recognized localized patterns in suicide methods and their relationship to specific physical spaces. Evaluating such place-based interventions, especially in the Circumpolar North, is challenging due to scale, funding, and ‘small numbers’ for the main outcomes [21,26]. As a consequence, the impact of many locally-developed programs focused on suicide remains uncertain [21], as in the examples above. Not knowing the impact makes it difficult to improve community responses to suicide and scale-up effective interventions to reach other settings and regions. 

Means restrictions approaches to suicide prevention often intersect with place and the built environment. However, generating timely and localized knowledge about specific locations and methods of suicide deaths is difficult with administrative and vital statistics data [6]. The research agenda proposed by Kariippanon and colleagues provides innovative directions for research and interventions related to means restriction in the built environment [6]. I would like to suggest that their agenda may be further strengthened by paying attention to specific methods, along with place and location. 

In practical terms, this could involve extracting and analyzing detailed data on suicide deaths from coroner and medical examiner records, and on suicide attempts from clinical or surveillance data. To this end, data collection should include information about housing or building type (hotel, motel, social housing, shed/garage, private residence/house), room (bedroom, bathroom, basement), institutional setting (hospital, school, prison), specific outdoor environments (river, bridge, road), in addition to more details about ligatures and anchor points and type of firearm or poison. Place and the built environment are already underlying dimensions of public health approaches to suicide prevention. Efforts to increase what population data can reveal about these dimensions are necessary to help uncover new directions for prevention. 
